# Biomimetic Stator Vane Design for Radial Turbines in Waste Heat Recovery Applications

**DOI:** 10.3390/biomimetics10070463

**Published:** 2025-07-15

**Authors:** Fuhaid Alshammari, Ibrahim Alatawi, Muapper Alhadri

**Affiliations:** Mechanical Engineering Department, Engineering College, University of Hail, Hail 2440, Saudi Arabia; ib.alatawi@uoh.edu.sa (I.A.); m.alhadri@uoh.edu.sa (M.A.)

**Keywords:** Organic Rankine Cycle (ORC), biomimetic, radial inflow turbine, stator vane camber, turbine efficiency, nature-inspired engineering

## Abstract

Organic Rankine Cycle (ORC) systems are widely used for converting low-temperature waste heat into useful power, but their overall efficiency depends heavily on the turbine’s performance, particularly the stator vane design in radial turbines. This study introduces a biomimetic approach to turbine design by implementing cambered stator vanes inspired by bird feather geometry. These specially shaped vanes are added to a radial inflow turbine and compared to a traditional design that uses straight (symmetric) vanes. The new cambered design helps guide the airflow more effectively, leading to higher tangential speeds and better energy transfer. Simulations show that this design increases the turbine’s power output from 388.6 kW to 394.87 kW and improves the system’s overall efficiency from 8.78% to 10.12%. A detailed study of different camber levels found that moderate curvatures (around 8–12%) gave the best results. Overall, this study demonstrates that implementing biomimetic cambered stator vanes in radial turbines can significantly enhance turbine performance and improve cycle-level efficiency in ORC systems for waste heat recovery.

## 1. Introduction

Low-temperature heat sources, like industrial waste heat, offer a big but underused opportunity for generating clean power [1]. However, traditional steam-based systems do not work efficiently at these lower temperatures [2]. The Organic Rankine Cycle (ORC) offers a promising alternative by employing organic working fluids with lower boiling points, making it more suitable for low-temperature heat recovery applications [3,4]

In Organic Rankine Cycle (ORC) systems, the turbine is one of the key components that has a major impact on how efficiently the system runs, how much power it produces, and how stable it is during operation [5]. Among the different types of turbines, radial inflow turbines are commonly used in small- to medium-scale ORC setups [6] because they are compact, deliver high power output for their size, and perform well under changing conditions [7]. They are especially effective for tapping into low-temperature heat sources like industrial waste heat [8] or geothermal energy [9] thanks to their ability to manage large energy drops in just one stage [10]. That said, the turbine’s internal design, particularly the shape and layout of the stator vanes, plays a huge role in how smoothly the flow moves through the system and how much energy is lost [11]. Even small design improvements can make a big difference in how well the turbine and the entire ORC system perform, which is why improving turbine design remains a top priority in efforts to make waste heat recovery more efficient [12].

However, standard radial turbines struggle under high-pressure conditions or when running outside their ideal operating conditions [13]. Nature often solves similar problems efficiently [14]. For example, trees and animals use branching flow networks to control pressure and reduce energy loss. Termite mounds [15] and plant leaves manage heat using smart shapes and surface designs [16]. In recent years, nature-inspired solutions have extended beyond aerodynamic shapes to include optimization algorithms and adaptive mechanisms. Bio-inspired optimization techniques, such as genetic algorithms, particle swarm optimization, and artificial immune systems, have been employed in turbomachinery design to mimic natural selection and adaptation processes, enabling efficient exploration of complex design spaces. Additionally, some studies have explored rotating mechanisms inspired by insect wings or fish propulsion to enhance energy conversion in specialized fluid dynamic systems. Inspired by these examples, this study applies the idea of staged pressure drops, like in natural systems, to the design of radial turbines. The goal is to minimize energy loss, similar to how nature minimizes waste and inefficiency.

Many recent studies have supported this nature-inspired (biomimetic) approach. For instance, Regassa et al. [17] showed how the design of sunflowers and water plants can improve floating solar panels. Gad-eL-Hak [18] designed turbo-expanders and blade tips based on animals like harbor seals to reduce aerodynamic drag. However, the focus was on the aerodynamic forces and mechanical evaluation. Nabawy and Crowther [19] demonstrated the aerodynamic benefits of curved (cambered) bird wings, which is relevant for turbine blade shapes. Rojas-Galván et al. [20] presented a comparative analysis of bio-inspired optimization algorithms for enhancing artificial neural network (ANN)-based Maximum Power Point Tracking (MPPT) in photovoltaic systems under partial shading. Their study focused on renewable energy forecasting rather than designing an energy recovery system. Chen et al. [21] introduced a hybrid whale optimization algorithm for improving the aerodynamic performance of turbine blades by mimicking fish schooling behavior and bio-structural coordination. Their model demonstrated enhanced flow characteristics and pressure distribution across blade surfaces, confirming the potential of nature-inspired designs for improving fluid machinery. Wen et al. [22] focused on biomimetic trailing edge design in low-speed turbines, drawing inspiration from bird wings to reduce vortex shedding and drag. Their CFD analysis indicated that incorporating such bio-inspired geometries can substantially improve wake flow quality and overall aerodynamic performance. However, only trailing edge, which is part of rotor blade, was explored. Han et al. [15] reviewed 126 studies highlighting how biomimetic designs inspired by nature improve renewable energy systems, carbon sequestration, and energy storage for sustainability. Despite promising advancements, widespread adoption faces challenges related to high costs, scalability, and limited interdisciplinary integration. Bahambary et al. [23] reviewed recent advances in biomimetic wind energy systems, focusing on how natural structures like flapping wings, fish tails, and bird feathers inspire the design of efficient, low-noise, and adaptive turbines. They found that biomimetic wind energy systems can enhance aerodynamic performance, reduce noise, and improve energy capture efficiency, but face limitations in scalability, structural complexity, and integration with conventional turbine technologies. Pinzon et al. [24] investigated the potential of bio-optimizing renewable energy systems in buildings. The results were promising, demonstrating a 2% efficiency improvement when incorporated into the original pre-optimization system.

This study makes a distinct contribution to the field by introducing a biomimetic approach at the component level of energy systems, focusing specifically on stator vane geometry within radial inflow turbines used in Organic Rankine Cycles (ORCs). Unlike prior works that emphasized system-level integration of biomimetic concepts, this research implements biologically inspired design directly within the turbine’s aerodynamic structure. By mimicking the asymmetric camber of bird feathers, the study proposes a vane shape that enhances flow turning and tangential momentum, thereby improving energy transfer within the turbine. This work extends beyond conceptual design by embedding the biomimetic vanes into a complete ORC thermodynamic model and quantifying their effect on system-level performance. It demonstrates a measurable increase in turbine power output and thermal efficiency, confirming that aerodynamic improvements at the vane level can translate into significant thermodynamic gains. Unlike prior research, which primarily relies on computational optimization without directly evaluating the effect of shape on cycle performance, this approach delivers clear numerical validation of the benefits. Table 1 summarizes the critical geometric, flow, and thermodynamic parameters evaluated in this study to assess the impact of biomimetic stator vane design on ORC turbine performance. These parameters include vane geometry features such as camber angle, chord length, and blade pitch, as well as flow behavior metrics like turning angle and Mach number. Additionally, key performance indicators, such turbine power, isentropic efficiency, and cycle thermal efficiency, are used to quantify the system-level impact of vane shape modifications. This structured overview provides clarity on the scope of the parametric study and supports the comparative analysis between cambered and un-cambered vane designs.

## 2. System Modelling

**Figure 1:**
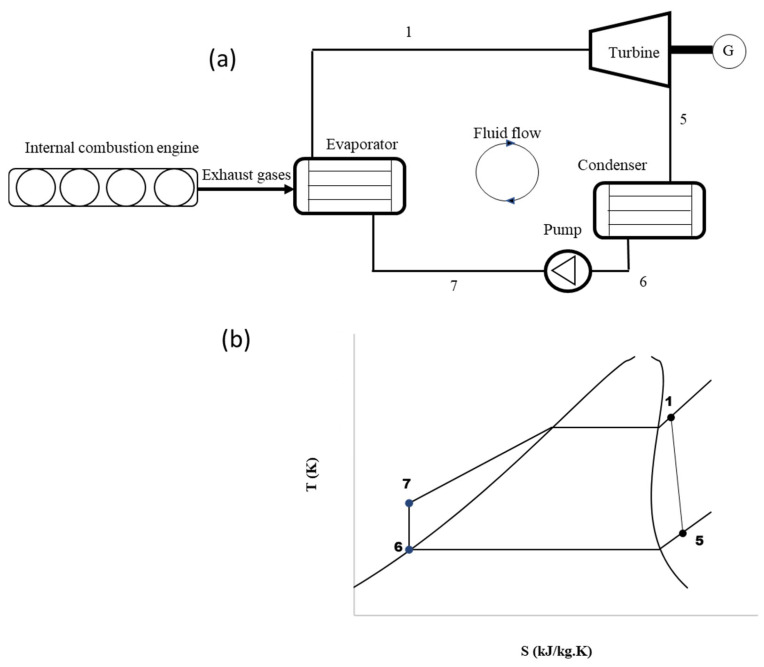
(**a**) Typical organic Rankine system coupled to an internal combustion engine, (**b**) Temperature-entropy diagram of the ORC system.

This section explains how the Organic Rankine Cycle (ORC) system was modeled using a steady state one dimensional thermodynamic approach, including a radial inflow turbine inspired by natural designs. Figure 1a represents the schematic diagram of the ORC system, while Figure 1b shows the T–s diagram (Temperature vs. Entropy). This method has been widely adopted in the literature for its ability to provide rapid, system-level insights into component interactions. It has been proven especially effective for preliminary design evaluations. The ORC is intended to recover waste heat from a heavy-duty diesel engine used at the Hail Electricity Company. This engine is a 10 L standby stationary diesel unit that powers auxiliary equipment. It typically produces exhaust gases at around 320 °C. These hot gases are directed to the ORC’s evaporator, where they heat and vaporize the working fluid, R245fa in this case. The details of the ORC model are described in the next subsection. It is worth noting that Stations 1 to 5 represent the turbine stage as shown in the previous study [25]. The volute inlet, stator vane inlet, rotor inlet, and rotor exit are denoted as Stations 1, 2, 3, and 4, respectively.

Before presenting the governing equations, the following assumptions were made to simplify the thermodynamic modeling of the ORC system. These assumptions are consistent with standard practices in steady-state ORC simulations and ensure tractability while maintaining accuracy in performance prediction:

- Steady-state operation: The system is assumed to operate under constant thermal and mechanical conditions throughout the cycle.
- Negligible pressure drops in pipes and heat exchangers: Pressure losses in piping and auxiliary components are neglected to focus on major thermodynamic performance contributors.
- No heat loss to surroundings: It is assumed that all heat exchanges occur within the defined system boundaries, with no environmental heat leakage.
- Working fluid in thermodynamic equilibrium (R245fa): The fluid properties are calculated under equilibrium conditions using reliable thermodynamic property databases.
- Isentropic efficiency for pump: Pump efficiency is assumed to be 70% to account for deviations from ideal behavior.

### 2.1. Cycle Modelling

The ORC system is analyzed using energy balance equations applied to each component, with the key states shown in Figure 1. To keep the analysis straightforward, heat losses in the components and connecting pipes are ignored. It is also assumed that all heat exchangers work at constant pressure. The amount of heat added to the system through the evaporator is calculated using Equation (1).

(1) $${\dot{Q}}_{E}=\dot{m}\left(h_{1}-h_{7}\right)$$

Turbine power is calculated using Equation (2). It can also be determined using Equation (3) [26], which is particularly important because it links the turbine’s power output to the tangential velocity of the flow. This velocity is influenced by the shape of the turbine vanes—whether they are cambered or un-cambered—making vane design a key factor in performance.

(2) $${\dot{W}}_{T}=\dot{m}\left(h_{1}-h_{5}\right)$$

(3) $${\dot{W}}_{T}=\dot{m}\left(U_{5}C_{\theta 5}-U_{1}C_{\theta 1}\right)$$

The fluid leaving the condenser is assumed to be a saturated liquid to ensure the pump operates efficiently. The amount of heat rejected by the condenser is calculated using Equation (4).

(4) $${\dot{Q}}_{C}=\dot{m}\left(h_{5}-h_{6}\right)$$

The power consumed by the pump to raise the fluid pressure from the condenser level to the evaporator level is calculated using Equation (5).

(5) $${\dot{W}}_{p}=\dot{m}\left(h_{7}-h_{6}\right)$$

The overall performance of the cycle is evaluated using net power output and thermal efficiency, which are calculated using Equations (6) and (7), respectively.

(6) $${\dot{W}}_{net}={\dot{W}}_{Turb}-{\dot{W}}_{Pump}$$

(7) $$\eta_{th}=\frac{{\dot{W}}_{net}}{{\dot{Q}}_{E}}$$

### 2.2. Turbine Modelling

**Figure 2:**
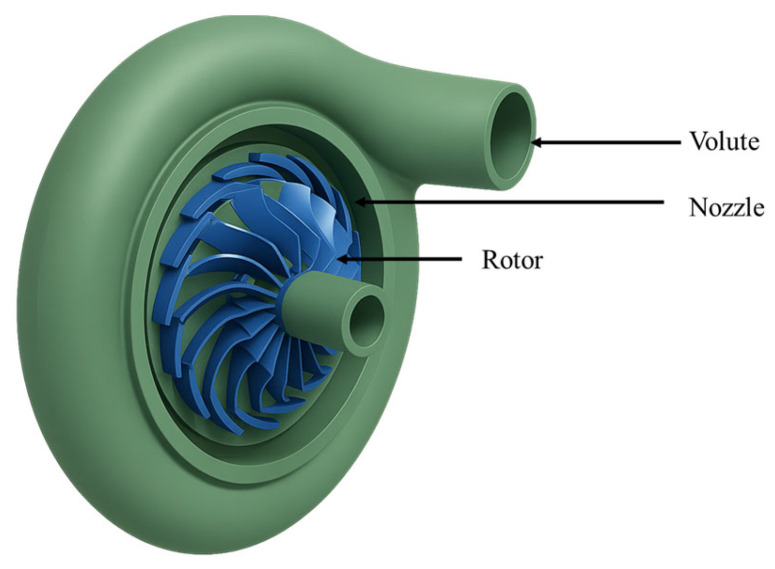
Components of a radial inflow turbine.

Radial inflow turbines play a key role in Organic Rankine Cycles (ORCs) by converting heat from organic fluids into useful mechanical power. As shown in Figure 2, these turbines typically consist of three main parts: the volute, nozzle, and rotor. The volute serves as the inlet, helping to evenly distribute the fluid around the turbine. The nozzle then speeds up the fluid, turning heat into kinetic energy and directing it onto the rotor blades. Finally, the rotor slows down the fluid and captures its energy, turning it into rotational motion. A more detailed model of the turbine can be found in a previous study by one of the authors [25].

**Figure 3:**
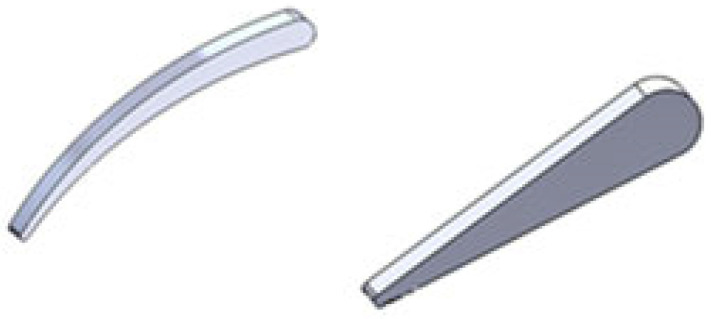
Three-dimensional CAD models of stator vane geometry: (left) biomimetically inspired cambered vane and (right) traditional un-cambered vane. The cambered profile mimics the natural curvature of bird feathers to enhance flow turning and energy transfer within the turbine stage.

**Figure 4:**
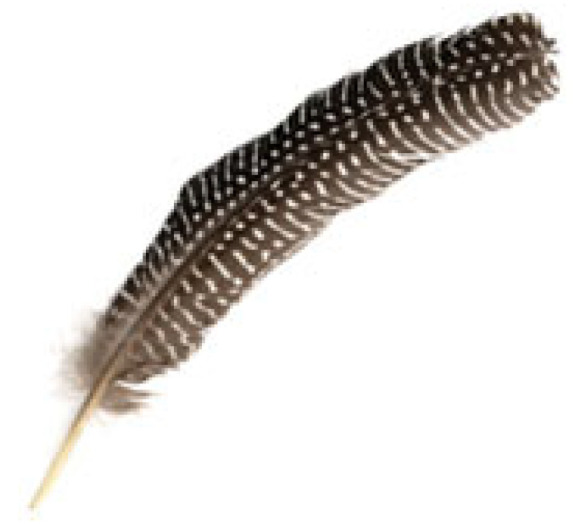
Shape of a turkey feather used as biomimetic inspiration for cambered stator vane design. The natural curvature and asymmetric camber of the feather provide flow-directing characteristics analogous to aerodynamic turning in turbine vanes.

Small radial inflow turbines often use un-cambered (symmetric) stator vanes, as shown in Figure 3, because they are easier and cheaper to design and manufacture, an important factor for small-scale systems that aim to be cost-effective. However, in nature, structures like turkey feathers are rarely symmetric. As shown in Figure 4, turkey feathers typically have a cambered (curved) shape, which plays a major role in improving aerodynamic performance during flight, especially in short bursts, gliding, and quick turns.

The camber shape changes how air flows over the feather. Air moves faster over the curved upper surface, creating lower pressure, while the slower air under the flatter lower surface results in higher pressure. This pressure difference produces lift helping birds like turkeys take off quickly with just a few wingbeats [27]. In addition, camber increases the average airflow over the top, which helps keep the flow attached and delays separation, resulting in better lift compared to flat or symmetric feathers. Cambered feathers also reduce pressure drag by streamlining the wing and allowing smoother recovery of airflow at the trailing edge [28]. This is especially useful during low-speed flight, where pressure drag is the main source of resistance. While the added curvature does slightly increase surface area, and potentially skin friction, the overall drag usually decreases. That is because cambered shapes keep the airflow more organized and reduce turbulence, leading to less energy loss. Finally, this aerodynamic efficiency is key to how turkeys fly. Their short, powerful flights rely on generating strong lift with minimal energy, and the cambered shape helps improve the lift-to-drag ratio. This makes each wingbeat more effective, reducing wasted effort and helping the bird move quickly and efficiently.

The curved shape of a turkey feather can inspire better aerodynamic design in turbine nozzle vanes. As shown in Figure 3, adding camber to a traditionally symmetric nozzle vane may improve how air flows over it. This is especially important in radial inflow turbines operating at high pressure ratios, where aerodynamic performance becomes more challenging. Cambered vanes offer a better lift-to-drag ratio, which helps the turbines stay efficient even under tough conditions. Their curved shape allows the vane to generate more lift while reducing drag, which is critical when dealing with fast-moving, high-pressure flows. These vanes also help prevent or delay flow separation, a common problem in high-pressure turbines where airflow changes direction sharply and pressure drops quickly. In addition, cambered vanes are more effective at managing sharp flow turns, making them more stable and reliable under high-pressure gradients. They perform well in high-speed flows by minimizing drag and improving how much energy can be extracted from the fluid. All of these benefits make cambered vanes a smart choice for boosting the efficiency and performance of radial inflow turbines.

As shown in Equation (3), turbine power is directly related to the tangential velocity of the flow. This velocity depends heavily on the vane’s turning angle ∆θ, which is controlled by the amount of camber in the stator vane as shown in Equation (8). In the case of symmetric (zero camber) vanes, the turning angle is small, which leads to lower swirl in the flow and less torque applied to the rotor. On the other hand, cambered vanes create a larger turning angle, allowing the stator to add more tangential momentum to the fluid. This increase in momentum enhances the rotor’s ability to extract energy. By examining the velocity triangle at the stator exit, the tangential component of the absolute velocity can be calculated using an Equation (9). where $C_{1}$ is the absolute velocity of the flow. As the camber increases, so does the turning angle, more efficient energy transfer to the rotor takes place. Substituting this into the simplified Euler equation, Equation (10) is obtained.

(8) $$∆\theta =\alpha_{out}-\alpha_{in}$$

(9) $$C_{\theta 1}=C_{1}\cos\left(∆\theta \right)$$

(10) $${\dot{W}}_{T}\propto U_{1}C_{1}\cos\left(∆\theta \right)$$

### 2.3. Stator Vane Modelling

**Figure 5:**
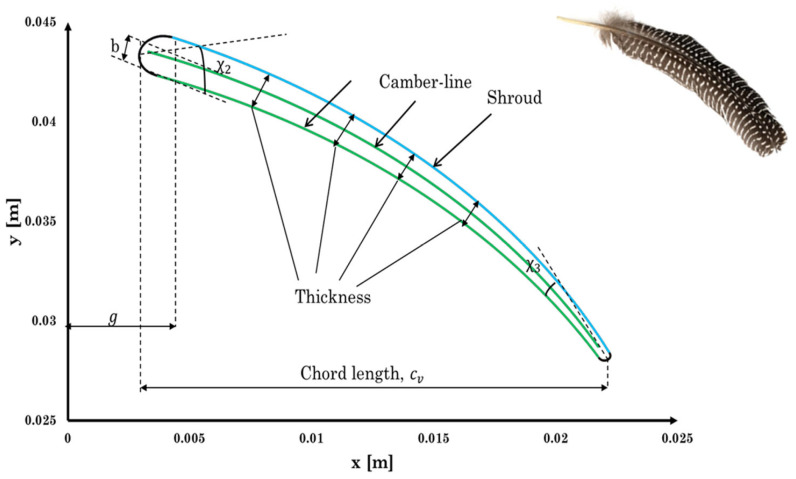
Overlay of the cambered stator vane geometry with the natural curvature of a turkey feather. The structural alignment highlights the aerodynamic similarity between biological and engineered shapes, demonstrating how the feather-inspired camber profile was adopted to enhance flow turning and efficiency in the ORC turbine design.

To minimize incidence losses, the nozzle vanes should be set at an appropriate blade angle to ensure the flow enters the rotor smoothly with the correct swirl direction. The vane thickness is distributed along a parabolic camber line, following the method described in Aungier [29]. The governing equation for the parabolic-arc camber line is provided in Equation (11), with the coordinates (*x*, *y*) illustrated in Figure 5. Based on the value of x, the corresponding stator geometry parameters can be determined. The required parameters are chord length $c_{v}$, maximum camber b, and its location g. The leading $\chi_{2}$ and trailing $\chi_{3}$ edge blade angles can be obtained using Equations (12) and (13). The angle of the blade comber-line is then the sum of the two blade angles.

(11) $$x^{2}+\frac{c_{v}-2g}{b}xy+\frac{{\left(c_{v}-2g\right)}^{2}}{{4b}^{2}}y^{2}-c_{v}x-\frac{{c_{v}}^{2}-4gc_{v}}{4b}=0$$

(12) $$tan\chi_{2}=\frac{4b}{\left(4g-c_{v}\right)}$$

(13) $$tan\chi_{3}=\frac{4b}{\left(3c_{v}-4a\right)}$$

**Figure 6:**
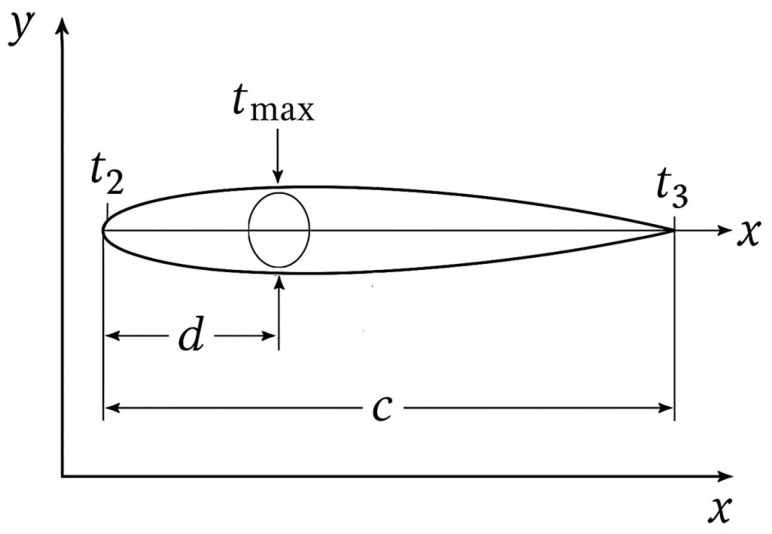
Geometrical parameters of the cambered vane profile. The chord length. The parameters are used to construct the vane’s thickness distribution and define its aerodynamic shape.

Aungier [29] introduced a set of equations, shown in Equations (14)–(18), to calculate blade thickness at any point along the camber line. $t_{max}$ and d are the maximum blade thickness and its location, respectively, as shown in Figure 6. $t_{2}$ and $t_{3}$ are the leading and trailing edge thicknesses.

(14) $$t=t_{ref}+[t_{max}-t_{ref}]\xi^{e}$$

(15) $$t_{ref}=t_{2}+[t_{3}-t_{2}](\frac{x}{d})$$

(16) $$\xi =\frac{x}{d}\;\mathrm{for}\;x\leq d$$

(17) $$\xi =\frac{c-x}{c-d}\;\mathrm{for}\;x>d$$

(18) $$e=[0.95\left(1-\frac{x}{c}\right)\left(1-\xi \right)+0.05]\sqrt{\frac{0.4d}{c}}$$

**Figure 7:**
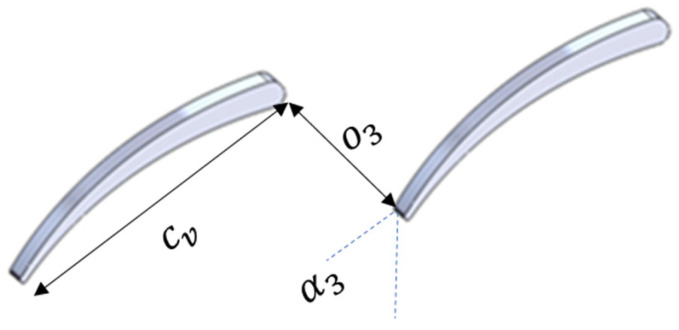
Parameters required to construct an adjacent vane.

The nozzle throat width, denoted as $o_{3}$ and shown in Figure 7, is a key parameter used to determine the spacing between adjacent vanes. It is calculated using Equation (19). If the flow at the stator exit is supersonic ($M_{3\;}\geq 1$), the throat width is determined using the mass continuity equation as shown Equation (19) to match the mass flow between the throat and the exit section. In this case, $\rho_{*}{\;\mathrm{and}\;a}_{*}$ represent the density and speed of sound under sonic conditions. The flow angle at the stator exit is $\alpha_{3}$ and $C_{m3}$ is the absolute velocity component in the meridional direction.

(19) $$\left\{\begin{array}{c} o_{3}=s_{v}sin\alpha_{3}\;\;\;for\;\;\;M_{3}<1\\ o_{3}=\frac{C_{m3}\rho_{3}}{\rho_{*}a_{*}}\;\;\;for\;\;\;M_{3}\geq 1 \end{array}\right.$$

## 3. Results and Discussions

### 3.1. Development of the Vane Geometry

Incorporating a biomimetic design inspired by bird feathers resulted in significant changes to the vane geometry, especially in the camber profile. In nature, feathers have an asymmetric, smoothly curved form that allows air to flow efficiently over the surface with minimal separation. Following this principle, the cambered vane used in this study was designed with an optimized curvature to enhance flow deflection and reduce incidence losses at the rotor inlet.

**Figure 8:**
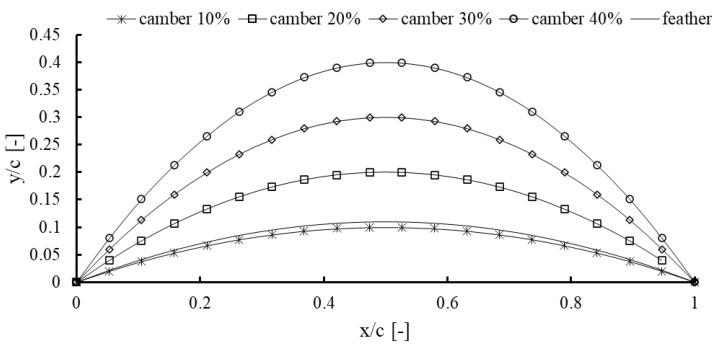
Comparison of camber line profiles for various vane camber percentages (10% to 40%) overlaid with the extracted camber distribution of a turkey feather.

As shown in Figure 8, the 10% camber profile closely mimics the natural curve of turkey flight feathers, making it both biologically inspired and aerodynamically efficient for use in vane design. This level of camber strikes a good balance as it provides enough lift while maintaining smooth, stable airflow, thanks to its parabolic shape and smooth pressure gradient.

Although higher camber values (such as 40–60%) can generate stronger pressure differences and potentially more lift, they come with trade-offs. These include sharper flow turning, a greater chance of boundary layer separation, and increased pressure drag, especially when operating off-design or at low Reynolds numbers. Such extreme camber can lead to unstable flow, poor energy conversion, and added complexity in both manufacturing and durability. That is why the 10% camber stands out as a practical and effective choice, offering a good compromise between performance, stability, and ease of implementation.

**Figure 9:**
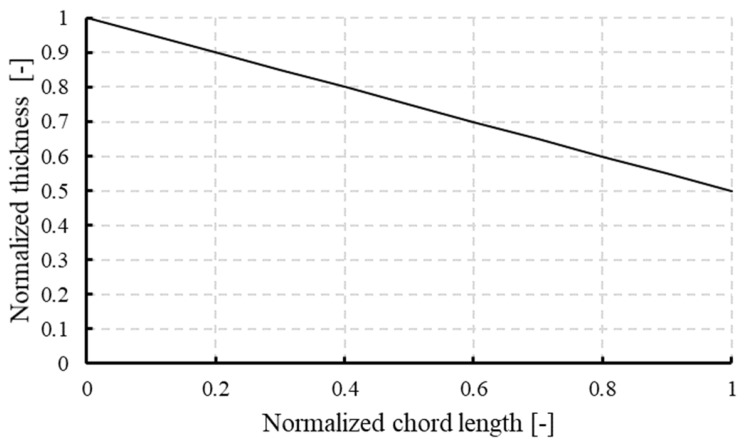
Normalized thickness distribution along the vane chord length. The thickness decreases linearly from the leading edge to the trailing edge, providing structural strength at the front and aerodynamic tapering at the rear.

Figure 9 shows the normalized thickness distribution along the vane’s chord length, displaying a linear taper from the leading edge to the trailing edge. The thickness starts at a maximum normalized value of 1.0 at the front and gradually decreases to about 0.45 at the rear. This tapering serves both aerodynamic and structural purposes. A thicker leading edge provides greater mechanical strength and better resistance to the impact of incoming flow, while a thinner trailing edge helps reduce wake formation and pressure drag. This design also draws inspiration from nature such as the tapering found in bird feathers and fish fins, where the smooth transition helps maintain attached flow and minimizes separation.

### 3.2. Case Study and Validation of the Model

To enable a clear and controlled comparison between cambered and un-cambered stator vanes, this study adopted the Organic Rankine Cycle (ORC) system layout originally presented by Sauret and Gu [30] as the baseline model. Their setup features a small-scale radial inflow turbine with symmetric (un-cambered) stator vanes, making it well-suited for direct performance benchmarking. In this work, all operating conditions, working fluid properties, and geometric parameters from the original model were kept unchanged to ensure consistency. The only modification was the replacement of the symmetric vanes with biomimetic cambered vanes. The operating conditions of the system are 413 K (turbine inlet temperature), 17.24 kg/s (mass flow rate), 24,250 rpm (rotational speed).

**Figure 10:**
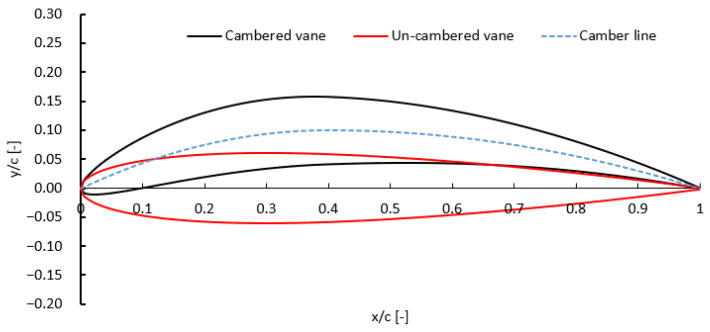
Geometric comparison between cambered and un-cambered stator vanes.

This targeted change allowed the study to isolate and assess the effects of camber-induced flow turning on turbine behavior, focusing specifically on tangential velocity, isentropic efficiency, and net power output. Table 2 highlights the main geometric differences between the two vane designs. The cambered vanes required a slightly longer chord length (about 8.6% increase) and a smaller blade pitch to preserve the same throat area and mass flow rate. The final vane shapes for both configurations are shown in Figure 10. The selection of the optimal chord length was guided by a parametric study, which is discussed in a later section.

A comparative analysis was carried out to assess how vane camber affects turbine and cycle performance. First, the un-cambered vane designed by the current model was validated with the one available in Sauret and Gu [30] to evaluate the robustness of the current model. As shown in Table 3, the results from the current un-cambered model closely align with those reported by Sauret and Gu [30], with turbine power differing by less than 0.1% and efficiency within 0.2%. A maximum deviation of 1.7% is obtained with a Mach number which is still acceptable. This strong agreement confirms the accuracy of the current simulation framework and its suitability for further analysis involving cambered vanes.

When the un-cambered vane was replaced with a biomimetic cambered design, significant improvements in aerodynamic and thermodynamic performance were observed. The exit Mach number increased from 0.865 to 1.12, indicating stronger flow acceleration and more effective energy extraction. The exit enthalpy dropped from 476.5 kJ/kg to 432.2 kJ/kg, confirming greater conversion of thermal to kinetic energy. Notably, the axial velocity component at the vane exit rose sharply from 124 m/s to 287 m/s, highlighting the improved flow turning provided by the cambered geometry.

These aerodynamic gains translated into measurable system improvements: turbine efficiency rose from 76.94% to 77.5%, and the overall cycle thermal efficiency increased from 9.78% to 10.12%. This comparison clearly demonstrates the performance benefits of cambered vanes and supports the value of biomimetic design in enhancing waste heat recovery systems.

### 3.3. Parametric Study

**Figure 11:**
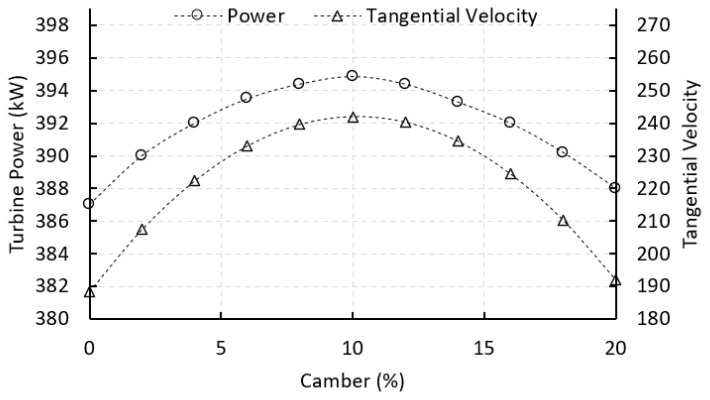
Effect of vane camber on turbine power output and tangential velocity. Both variables exhibit a parabolic trend, peaking at approximately 10% camber. Increased camber enhances flow turning and energy transfer up to the optimal point, after which performance declines due to over-deflection.

Figure 11 shows the results of a parametric study evaluating how different levels of stator vane camber affect the tangential velocity component $C_{\theta }$ and the resulting turbine power output. Both curves follow a clear parabolic trend, rising with camber up to a certain point and then declining. As camber increases from 0% to around 10%, the added curvature strengthens the turning of the working fluid. This causes the flow to deflect more from the axial toward the tangential direction, increasing $C_{\theta }$, which is the key component driving torque on the rotor blades. Turbine power increases in step with this tangential velocity, in line with Equation (3). In this initial range, the enhanced flow turning from the added camber outweighs the small reduction in the cosine of the turning angle. The result is a more effective transfer of energy to the rotor. Maximum power is achieved at around 10% camber, reaching 394.87 kW, representing an optimal balance between flow redirection and aerodynamic loading.

Beyond this point, however, additional camber starts to produce diminishing returns. The flow exits the vane at increasingly steep angles, which, although they may slightly boost absolute velocity due to stronger expansion, cause a drop in the useful tangential component $C_{\theta }$. This decline is mainly due to the nonlinear decrease in cos(Δθ), as the flow becomes less aligned with the rotor’s tangential direction. Excessive camber can also lead to greater profile losses and possible flow separation, which further reduces performance. The drop in both tangential velocity and turbine power at high camber levels highlights the importance of carefully optimizing vane geometry. The results show that moderate camber, particularly in the 8% to 12% range, offers the best aerodynamic performance, maximizing energy extraction while keeping losses low. This finding also supports the biomimetic design approach as it mirrors the aerodynamic efficiency seen in bird feathers, especially in species that rely on quick, powerful bursts of flight.

**Figure 12:**
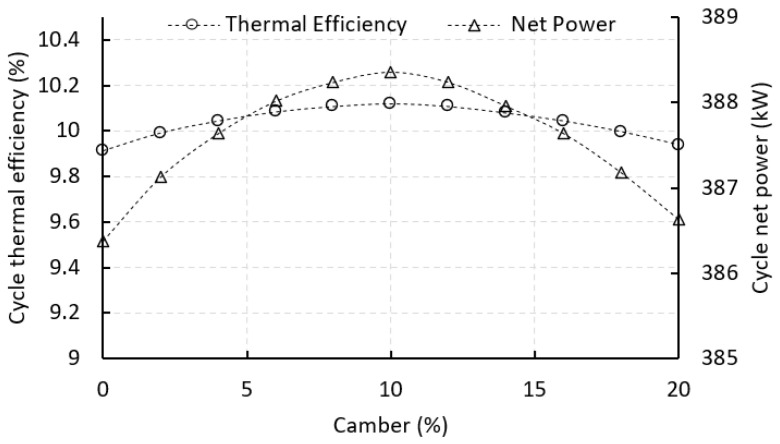
Effect of vane camber on cycle thermal efficiency and net power output.

Figure 12 illustrates how ORC thermal efficiency and net power output vary with changes in stator vane camber. Both follow a parabolic trend, peaking at around 10% camber, but they respond differently to the variation. Net power output shows a sharper change, rising from about 386.5 kW at 0% camber to a peak of 388.36 kW at 10%, before dropping back to roughly 386.7 kW at 20%. In comparison, thermal efficiency changes gradually, increasing from 8.78% to 10.12% over the same camber range.

This difference is due to how camber impacts mechanical energy extraction in the turbine versus the overall thermodynamic cycle. Increasing camber improves flow turning in the stator, which boosts the tangential velocity at the rotor inlet and increases turbine power. Since net power is directly related to this mechanical output, after accounting for losses like those from the pump, it reacts strongly to changes in vane geometry.

Thermal efficiency, however, depends on more than just the turbine. It is influenced by the heat input and other components in the cycle, such as the pump and evaporator. So, while the turbine power may improve significantly with camber, the overall gain in efficiency is more modest because the heat input remains relatively stable. This causes the efficiency curve to rise more gently compared to the sharper response in net power.

**Figure 13:**
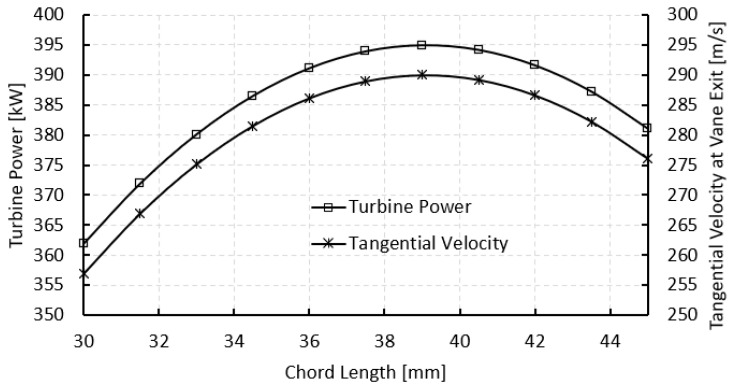
Effect of chord length on turbine power and tangential velocity.

The chord length of stator vanes is a critical factor in shaping the aerodynamic behavior and energy conversion efficiency of a radial inflow turbine. As shown in Figure 13, turbine power output increases with chord length up to a peak near 39.1 mm, matching the cambered vane configuration used in this study. This initial rise is due to improved flow control and redirection offered by longer vanes. A larger chord provides more surface area for pressure development, allowing the vane to impart a stronger tangential force to the working fluid.

As the chord length increases, the vane can more gradually and effectively steer the flow from an axial direction to a more tangential one at the exit. This boosts the turning angle and in turn increases the tangential velocity component, which directly contributes to higher torque on the rotor and greater turbine power. However, this improvement has a limit. Beyond the optimal point, further increasing the chord leads to diminishing returns, and eventually to a slight decline in performance. Very long vanes reduce the pitch-to-chord ratio, increasing blockage and restricting the flow passage. This can lower the effective flow coefficient and reduce the tangential velocity, ultimately decreasing the turbine’s power output. Based on this analysis, a chord length of 39.1 mm was identified as the optimal value, as summarized in Table 2.

## 4. Conclusions

This study introduced a biomimetic-inspired design for radial turbine stator vanes by implementing cambered vane geometries modeled after bird feather structures. A validated 1D thermodynamic model was used to evaluate the impact of vane camber on the performance of an Organic Rankine Cycle (ORC) system. The comparison between uncambered and cambered vanes revealed that cambering increases the turning angle, improves flow guidance, and leads to higher turbine power output and cycle thermal efficiency. Parametric studies on camber percentage, blade pitch, and chord length further demonstrated that aerodynamic improvements in the vane geometry significantly influence tangential velocity, Mach number, and expansion efficiency. The major contributions of this work include:

- Demonstrating a practical approach to integrating biomimetic concepts into radial turbine design using a low-complexity thermodynamic model. Implementation of biomimetic cambered vanes increased the turbine power output from 388.71 kW (uncambered) to 394.87 kW (cambered) under the same inlet conditions.
- Establishing a direct correlation between vane camber characteristics and turbine/cycle performance. Turbine and thermal efficiencies, respectively, increased from 76.94% and 8.78% (with un-cambered vanes) to 77.5% and 10.12% (with cambered vanes), attributed to improved flow alignment and reduced aerodynamic losses.
- Increasing exit Mach number from 0.865 to 1.12, showing better utilization of nozzle area and effective flow acceleration due to cambering.
- Improving tangential velocity trends, where optimized vane camber and pitch produce up to 12% higher values, further supporting increased turbine work.

As a direction for future research, detailed Computational Fluid Dynamics (CFD) and Finite Element Analysis (FEA) will be carried out to validate the aerodynamic performance and assess the structural integrity of the cambered vane under realistic flow and loading conditions. These advanced simulations will offer deeper insights into local flow behavior, stress distribution, and potential deformation, supporting the refinement of biomimetic vane designs for practical implementation.

## Figures and Tables

**Table 1:** Key parameters analyzed in the biomimetic radial turbine study.

Category	Parameter	Description
Geometric Design	Camber Angle	Degree of curvature applied to the vane centerline
	Chord Length	Distance from leading to trailing edge of the vane
	Vane Pitch	Spacing between adjacent vanes
	Vane Thickness	Thickness distribution along the vane.
Flow Characteristics	Flow Turning Angle	Angle through which the flow is redirected by the vane
	Exit Mach Number	Flow speed at vane exit normalized by local speed of sound
Thermodynamic Performance	Turbine Power Output	Net shaft power generated by turbine
	Isentropic Efficiency	Ratio of actual turbine work to ideal isentropic work
	Cycle Net Work	Net useful work of the ORC system
	Cycle Thermal Efficiency	Efficiency of converting thermal input to useful work

**Table 2:** Geometric comparison between un-cambered and cambered stator vane designs.

Parameter	Un-Cambered Vane Sauret and Gu [30]	Cambered Vane Current Model
Inlet radius (mm)	83.45	83.45
Exit radius (mm)	66.67	66.67
Chord length (mm)	35.2	39.1
Camber angle (°)	0	25.0
Throat area (mm^2^)	212	212
Blade pitch (mm)	16.8	15.3

**Table 3:** Performance comparison between un-cambered and cambered vane configurations.

Variable	Un-Cambered Vane (Sauret and Gu) [30]	Un-Cambered Vane Current Model (Validation with Sauret and Gu [30])	Error (%)	Cambered Vane Current Model
Mach number at vane exit [-]	0.88	0.865	1.70	1.12
Turbine efficiency [%]	76.8	76.94	0.18	77.5
Turbine power output [kW]	388.6	388.71	0.03	394.87
Vane inlet enthalpy [kJ/kg]	499.2	504.6	1.08	504.6
Vane exit enthalpy [kJ/kg]	-	476.5	-	432.2
Vane inlet pressure [MPa]	4.9896	4.994	0.09	4.994
Vane inlet velocity (axial) [m/s]	-	0	-	116
Vane exit velocity (axial) [m/s]	-	124	-	287
Cycle thermal efficiency [%]	-	8.78	-	10.12

## Data Availability

Data will be available upon request.

## Nomenclature

**Symbol**	**Description**	**Unit**	**Symbol**	**Description**	**Unit**
c	Absolute velocity	m/s	y/c	Non-dimensional thickness distribution	–
$C_{\theta }$	Tangential velocity at rotor inlet	m/s	U	Rotor blade speed	m/s
$c_{v}$	Chord length of stator vane	m	$t_{max}$	Maximum thickness of cambered vane	m
d	Location of max thickness along chord	m	t	Vane thickness at a given axial location	m
e	Camber shaping exponent	-	$t_{ref}$	Reference baseline thickness	m
h	Specific enthalpy	kJ/kg	${\dot{W}}_{net}$	Net cycle power output	kW
$\dot{m}$	Mass flow rate	kg/s	${\dot{W}}_{T}$	Turbine power output	kW
ORC	Organic Rankine cycle	–	α	Absolute flow angle	degrees (°)
P	Pressure	Pa or bar	∆θ	Turning angle due to vane camber	degrees (°)
R245fa	Organic working fluid		$\eta_{th}$	Cycle thermal efficiency	%
s	Specific entropy	kJ/kg·K	ξ	Normalized axial position along vane chord	-
T	Temperature	K	χ	Edge blade angles	degrees (°)
x/c	Non-dimensional axial coordinate	–			
